# Hodgkin Lymphoma—Review on Pathogenesis, Diagnosis, Current and Future Treatment Approaches for Adult Patients

**DOI:** 10.3390/jcm10051125

**Published:** 2021-03-08

**Authors:** Jesko Momotow, Sven Borchmann, Dennis A. Eichenauer, Andreas Engert, Stephanie Sasse

**Affiliations:** 1 German Hodgkin Study Group (GHSG), Department I of Internal Medicine, Center for Integrated Oncology Aachen Bonn Cologne Duesseldorf, Medical Faculty and University Hospital Cologne, University of Cologne, 50937 Cologne, Germany; jesko.momotow@uk-koeln.de (J.M.); sven.borchmann@uk-koeln.de (S.B.); dennis.eichenauer@uk-koeln.de (D.A.E.); A.Engert@uni-koeln.de (A.E.); 2 Department IV of Internal Medicine, Center for Integrated Oncology Aachen Bonn Cologne Duesseldorf, University Hospital Aachen, University of Aachen, Pauwelsstraße 30, 52074 Aachen, Germany

**Keywords:** classical Hodgkin lymphoma (cHL), nodular lymphocyte-predominant HL (NLPHL), diagnosis, stage-adapted combined modality treatment, stem cell transplantation, targeted treatment approaches, immunomodulatory treatment

## Abstract

Hodgkin lymphoma (HL) is a rare malignancy accounting for roughly 15% of all lymphomas and mostly affecting young patients. A second peak is seen in patients above 60 years of age. The history of HL treatment represents a remarkable success story in which HL has turned from an incurable disease to a neoplasm with an excellent prognosis. First-line treatment with stage-adapted treatment consisting of chemotherapy and/or radiotherapy results in cure rates of approximately 80%. Second-line treatment mostly consists of intensive salvage chemotherapy followed by high-dose chemotherapy (HDCT) and autologous stem cell transplantation (ASCT). Novel approaches such as antibody drug conjugates and immunomodulatory drugs have shown impressive results in clinical trials in refractory and relapsed HL and are now increasingly implemented in earlier treatment lines. This review gives a comprehensive overview on HL addressing epidemiology, pathophysiology and current treatment options as well as recent developments and perspectives.

## 1. Introduction

Hodgkin lymphoma (HL) is a rare neoplasm of the lymphatic system representing one of the most common cancers in young adults [1]. The disease is characterized by a low number of malignant cells deriving from B-lymphocytes and an extensive inflammatory microenvironment. This unique histopathological picture and its pathogenesis are still only partially understood. In some patients, Epstein–Barr virus (EBV) infection has to be regarded as a relevant factor in pathogenesis. Certain genetic factors and HIV infection have been described as independent risk factors [2,3,4,5].

Histopathologically, 95% of HL cases are classified as cHL including the subtypes nodular sclerosing, mixed cellularity, lymphocyte-rich and lymphocyte-depleted HL. In 5% of cases, NLPHL is diagnosed [6]. While cHL is characterized by the presence of CD30-expressing Hodgkin and Reed–Sternberg (HRS) cells surrounded by a variety of inflammatory cells, the malignant cells in NPLHL are termed lymphocyte predominant (LP) cells. They are positive for CD20 and lack CD30. These cells are surrounded by mature lymphocytes.

The treatment of HL has developed over the last few decades [7]. Approximately 80% of patients can be cured using a stage-adapted first-line treatment consisting of chemotherapy and/or radiotherapy [8,9].

The current first-line approaches include combined modality treatment as well as PET-adapted strategies. PET-adapted approaches as well as the implementation of the anti-CD30 antibody-drug conjugate Brentuximab Vedotin (BV) aim at reducing treatment-associated long-term toxicity while maintaining treatment efficacy.

However, about 20% of cHL patients suffer from relapse or primary progressive disease. Second-line treatment usually consists of high-dose chemotherapy (HDCT) followed by autologous stem cell transplantation (ASCT) for those patients under the age of 60 years. With this intensive treatment approach, about 50% of patients can be cured [10,11].

The poor outcome of cHL patients relapsing after HDCT and ASCT has improved with the introduction of BV as well as the immunomodulatory treatment approach with checkpoint inhibitors.

## 2. Epidemiology and Risk Factors

Hodgkin lymphoma has an incidence of 2–3 per 100,000 individuals per year. In addition to a disease peak in the third decade of life, there is a second peak in the age group over 60 years [12,13].

Various factors seem to favor the occurrence of HL. The significantly increased risk of identical twins strongly indicates the role of genetics in HL. A couple of polymorphisms in the genes regulating immunological functions have been described to be associated with an increased risk of HL [14].

The Epstein–Barr virus (EBV) is detected in nearly 45% of HL patients [15]. However, some subtypes, such as nodular sclerosing, rarely show the intracellular Epstein–Barr viral genome. Thus, an expired EBV infection seems to be a trigger mechanism for the development of HL in some patients, but is not sufficient for the development of HL alone [2,15].

HIV-positive persons generally have an increased risk of developing HL. The incidence of HIV-associated HL has increased with the improved immune competence achieved by Highly Active Anti-Retroviral Therapy (HAART), emphasizing the pathogenetic role of the inflammatory microenvironment [16,17].

The combination of genetic factors, habits in certain socioeconomic milieus and external influences such as viral infections appear to increase the risk of disease.

## 3. Pathophysiology

The pathophysiology of HL is increasingly being understood: cHL is a B-cell lymphoma of germinal center origin that has lost its B-cell phenotype [18,19]. HRS cells harbor clonal rearrangements of hypermutated, class-switched immunoglobulin genes resulting in nonfunctional immunoglobulin genes lacking the expression of the cell surface B-cell receptor [20]. In a healthy B-cell, this should lead to apoptosis; however, in HL, these cells appear to be “rescued” from apoptosis by additional oncogenic events [20,21].

In addition to continued intracellular survival and proliferation signaling, HRS cells require a peculiar cellular microenvironment (TME). In fact, this microenvironment makes up most of the HL lesions, while HRS cells account for only a few percent of the analyzed cells in the tumor lesion. The HL microenvironment consists of lymphocytes, granulocytes, eosinophils, mast cells, tumor-associated macrophages and fibroblasts. The percentage of these immune cell types varies dependent the histological subtype of cHL.

A deeper insight into the mechanisms by which HRS cells orchestrate their microenvironment and evade T-cell and natural killer (NK)-cell-mediated antitumoral immune response significantly contributed to the understanding of HL biology, providing new treatment approaches [22].

Genomic analyses of HRS cells have shown that certain genetic aberrations significantly contribute to their altered interaction with the inflammatory microenvironment. Reduced MHC class I or II presentation on the HRS surface, either by downregulation, loss-of-function mutations (B2M) or translocations (CIITA) [23,24] impair antigen presentation. Furthermore, HRS cells frequently harbor an increased copy number of genes located on chromosome 9p24.1 encoding programmed death receptor ligands PDL1 and 2. The interaction of PDL1/2 with PD1 is regarded as a relevant pathomechanism of T-cell exhaustion. In addition, the NK-cell mediated antitumoral immune response might be inhibited by the aberrant expression of MICA in HRS cells [25].

The complex interactions of HRS cells with their inflammatory environment are not completely understood. Topological analyses by means of multiplex immunofluorescence and digital image analyses revealed that HRS cells predominantly interact with surrounding PDL1+ macrophages and PD1+CD4+ T cells, while CD8+ T cells can rarely be found close to HRS cells. Mass cytometric analyses confirmed the role of a CD4+T cell predominant and Th1-polarized but also immunosuppressive microenvironment in cHL [26,27].

These observations strongly suggested the potential of the immunomodulatory approach of the checkpoint blockade in cHL.

## 4. Diagnosis and Staging

The diagnosis of HL involves a multistage process. The removal and histopathological analysis of a lymph node or punch biopsy of another affected organ is the method of choice for diagnosis. Thus, pathological expert review is recommended. A fine needle biopsy alone is only sufficient if sufficient material for histopathological diagnosis can be obtained [28].

Detailed history, clinical examination as well as imaging procedures including contrast enhanced CT (ceCT) and 18FDG-Positron emission tomography (PET/CT) are mandatory for initial staging [29,30]. Moreover, PET/CT is highly sensitive to detecting bone marrow involvement and allows omission of the bone marrow biopsy in the case of PET negativity [31,32,33].

**Figure 1:**
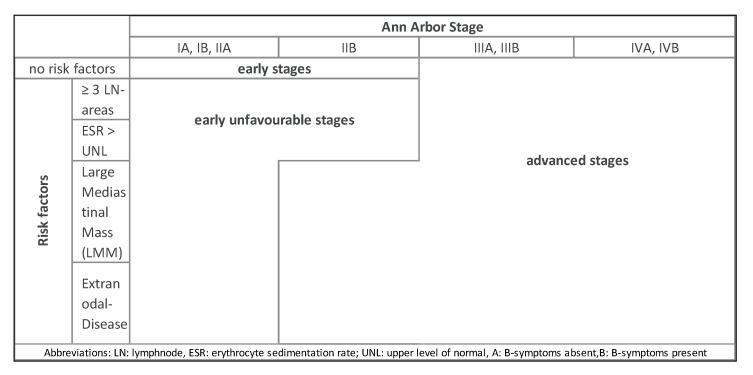
Risk-stratified staging according to risk factors and Ann Arbor stage.

Individual stages of HL are differentiated using the modified Ann-Arbor classification (Table 1) and defined risk factors. Risk stratification varies between Europe and North America (Table 2). In Europe, three different risk groups are relevant including early-stage favorable, early-stage unfavorable and advanced stage HL (Figure 1).

Before starting treatment, cardiovascular evaluation including ECG, echocardiogram, pulmonary function and thyroid hormone determination should be performed [35,36,37,38]. Since chemo- and radiotherapy can potentially affect the fertility of patients, all patients should be offered the possibility of fertility maintenance measures if family planning is not finalized [39,40].

## 5. Treatment Strategies

In the following chapter, we will discuss the current treatment approaches and recommendations for both subtypes, cHL and NLPHL. First-line treatment is discussed separately for early-stage favorable and unfavorable as well as advanced stage HL according to the risk stratification applied by GHSG and the EORTC/LYSA.

### 5.1. Classical Hodgkin Lymphoma (cHL)

#### 5.1.1. First-Line Treatment

##### Early-Stage Favorable cHL

Based on the results of the GHSG HD10, HD13 and HD16 trials, the current standard for early stage cHL (stage I/II without detection of any of the defined risk factors) consists of two cycles of doxorubicin, bleomycin, vinblastine, and dacarbazine (ABVD) and consolidation radiotherapy (“involved site” (IS) radiotherapy (RT) with 20 Gray (Gy)). Long time follow up analyses of the HD10 trial showed progression free survival rates (PFS) of 87% (HR, 1.0; 95% CI, 0.6–1.5) and overall survival rates (OS) of 94% (HR, 0.9; 95% CI, 0.5–1.6) for this treatment approach [9,41,42,43].

Despite these excellent treatment results, treatment-associated toxicity significantly contributes to long-term morbidity and mortality. [44]. In order to further reduce treatment-associated morbidity, more recent trials have evaluated response-adapted strategies and the reduction of chemotherapy intensity. Omitting parts of the ABVD regimen in the GHSG HD13 trial and the application of a PET/CT- response based radiotherapy approach in those patients achieving PET-negativity after two or three cycles of ABVD, respectively, within the GHSG HD16, H10 EORTC and RAPID-trial resulted in a significantly reduced tumor control [42,45,46]. Hence a PET-guided RT approach cannot be generally recommended so far, as maximum disease control is the main goal of therapy.

In the UK RAPID trial, 602 patients with stage I/IIA HL and no mediastinal bulk received three cycles of ABVD followed by PET/CT. Patients with a negative PET/CT (Deauville-score 1–2) were randomly assigned to receive 30 Gy Involved Field (IF)-RT or no further treatment. About 2/3 of patients enrolled had a favorable risk-profile according to the GHSG or EORTC risk classification. At 3-years, patients with a PET- negative PET/CT in the intent-to-treat and per-protocol cohorts had a PFS-difference of 3.8% (95% CI −8.8 to 1.3; 3-year PFS 94.6% vs. 90.8%) and 6.3% (95% CI −11.0% to 1.6%; 3-year PFS 97.1% vs. 90.8%) in favor of consolidative IF-RT in the intent-to-treat and per-protocol cohorts, respectively. In both analysis sets, the upper confidence interval limit exceeded the predefined noninferiority-margin for non-RT of 7%. Thus, the RAPID trial underlined the necessity for consolidating RT. Whether radiotherapy can be omitted in certain cases in order to avoid additional toxicity to the disadvantage of a better tumor control should be discussed individually with the patient.

##### Early-Stage Unfavorable cHL

Patients with initial diagnosis of early-stage unfavorable HL are usually treated with a combination of four cycles of polychemotherapy. Depending on response and intensity of systemic therapy, a consolidation radiotherapy is applied. Internationally, most groups applied four cycles of ABVD followed by 30 Gy RT as the treatment of choice for early unfavorable cHL [47,48,49]. With the advent of eBEACOPP (bleomycin, etoposide, doxorubicin, cyclophosphamide, vincristine, procarbazine, prednisone) in advanced stages, the randomized HD14 trial of the GHSG compared four cycles of ABVD with two cycles of eBEACOPP followed by two cycles of ABVD (“2+2”) and consecutive radiation with 30 Gy “involved field” RT (IFRT) [50]. The initial and the most recent follow-up analyses showed a significantly improved PFS with “2+2” over ABVD (91.2%; 95% CI, 89.0–93.4% vs. 85.6%; 95% CI, 82.9–88.4%). There was no difference in secondary primary malignancies and so far no significant overall survival difference has been documented [51].

Several trials aimed at reducing toxicity for patients while maintaining excellent tumor control by evaluating PET-driven treatment approaches. The recently completed GHSG HD17 trial showed that patients who are PET-negative after “2+2” do not require consolidating 30 Gy INRT radiotherapy (involved node RT). This implies that 84% of this group of patients no longer require consolidative radiotherapy and are adequately treated with chemotherapy alone [52]. Thus, a PET-guided “2+2” approach could also be a valid option for early-unfavorable cHL. In the EORTC H10 trial, those patients with a negative interim PET after two cycles ABVD, treated with four cycles of ABVD altogether, but without consolidating RT had an inferior PFS compared to those patients receiving 30 Gy IN-RT (5-year PFS rates 89.6% (95% CI, 85.5 to 92.6) vs. were 92.1% (95% CI, 88.0 to 94.8). Thus, the omission of consolidating radiotherapy in early-stage unfavorable HL can only be recommended after “2+2”, if maximum tumor control is intended.

In the EORTC/LYSA/FIL H10 trial an escalating approach was tested. Patients with early-stage unfavorable HL received two cycles of eBEACOPP after 2xABVD in case of a positive interim PET/CT after two cycles of ABVD. Although the pooled analysis included favorable and unfavorable patients, the 5-year PFS rates were 77.4% (95%CI, 70.4–82.9%) and 90.6% (95% CI, 84.7–94.3%) in the ABVD +INRT and eBEACOPP + INRT arms with a HR of 0.42 (95%CI, 0.23–0.74; *p* = 0.002) in favor of eBEACOPP. OS was 89.3% for ABVD + INRT and 96.0% for eBEACOPP + INRT, respectively.

The HR of 0.45 (95% CI, 0.19–1.07; *p* = 0.062) with regard to OS implies a benefit for a combination of ABVD with eBEACOPP in patients with a positive interim PET.

This data implies that in those patients with positive PET/CT after two cycles of ABVD consecutive treatment with two cycles of eBEACOPP and 30 Gy IS-RT should be recommended. [42].

##### Advanced-Stage cHL

Patients with advanced-stage cHL are more likely to relapse or have refractory disease and therefore require a more intensive treatment. Most patients in the advanced stages are usually treated with chemotherapy alone.

Internationally, treatment with six cycles of ABVD and consecutive PET-adopted radiotherapy of residual lesions or initial bulky disease with 30 Gy RT remains the standard therapy for advanced stages in most countries [48,53]. This is partly due to the management of the more toxic eBEACOPP, which requires an optimal medical infrastructure. However, the eBEACOPP regime results in more frequent and long-lasting remissions and the assumed increased risk of secondary primary malignancies has not yet been confirmed [54]. All studies directly comparing ABVD with eBEACOPP have shown that eBEACOPP therapy leads to a better overall survival and fewer relapses with a comparable rate of secondary neoplasia. A meta-analysis comprising almost 10,000 patients from 14 different studies comparing ABVD with eBEACOPP showed a significant survival benefit of 7% compared to ABVD in the advanced stages of HL [55]. Therefore, the treatment of advanced stage HL with eBEACOPP represents the standard of care by EORTC, LYSA and GHSG. In most Western countries, the eBEACOPP regime can be safely implemented in an inpatient and outpatient care setting. The recently updated NCCN guidelines emphasize the use of eBEACOPP in selected patients under the age of 60; however, they recommend an approach with ABVD upfront [56].

**Figure 2:**
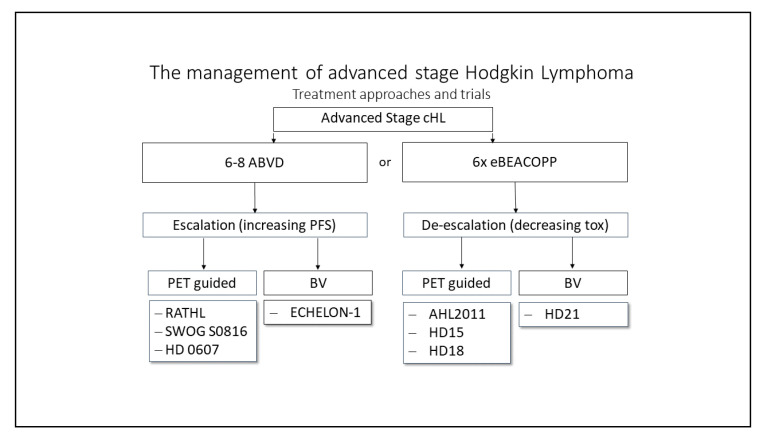
Treatment options overview for advanced classical Hodgkin lymphoma (cHL). The figure displays treatment approaches reviewed in clinical trials. Either escalating treatment to improve progression-free survival rates (PFS) by PET-guided switching to eBEACOPP or by addition of new drugs (BV) or de-escalation of efficient but toxic treatment approaches guided by PET/CT or including new targeted drugs.

As already mentioned in the previous segment, research groups aim at reducing toxicity for patients while maintaining tumor control, either by deescalating or escalating therapy regimens (Figure 2). Deescalating strategies aim at reducing chemotherapy toxicity during the course of the treatment, either by reducing the number of chemotherapy cycles or by adding new targeted treatment options to a modified treatment regimen. Escalating strategies imply an intensification of chemotherapy in case of insufficient tumor control; also, by either adding new targeted treatment options or switching to a more intensive treatment.

##### Deescalating Strategies

Based on the results of the GHSG HD18 study achieving a 5-year PFS of 90–92%, the current GHSG standard for patients in advanced stages under 60 years of age consists of four or six cycles of eBEACOPP—depending on the early PET-based response after the second cycle—followed by PET-based radiotherapy [57]. Thus, most patients with advanced cHL can be treated with 4x eBEACOPP alone. When given after two initial cycles of eBEACOPP, a de-escalation of the treatment by switching to ABVD appears be feasible, as shown by the LYSA AHL2011 trial [58].

The ongoing GHSG 21 trial evaluates a different approach to further reducing chemotherapy-associated acute and long-term toxicity in advanced stage cHL: the combination of BV with a modified eBEACOPP regimen termed BrECADD, which already showed promising results in a smaller randomized phase II trial [59].

##### Escalating Approaches

There is some discussion on PET-stratified approaches starting with ABVD. A small trial with 160 patients suggested a benefit for chemotherapy intensification with eBEACOPP for PET-positive patients after two cycles of ABVD [60]. However, the large SWOG-S0816 trial questions the sensitivity of an interim PET/CT after two cycles of ABVD. Although all patients in the SWOG S0816 trial with a negative PET after two cycles of ABVD, receiving four additional ABVD cycles, and those with a positive interim PET being subsequently treated with six cycles eBEACOPP achieved a similar 5-year OS, the relapse rate in those patients with negative interim-PET was close to 25% [61].

The Echolon-1 trial aimed improving tumor control in advanced cHL patients by adding the anti-CD30 immunoconjugate Brentuximab Vedotin to AVD. Patients were randomized to be treated with 6x ABVD or 6x BV-AVD. The trial showed an improvement in tumor control over ABVD, however also showing an increase in toxicity. The 2-year modified mPFS rates were 81.0% (95% CI, 77.6 to 83.9) in the A+AVD arm and 74.4% (95% CI, 70.7 to 77.7) in the ABVD Arm HR 0.72 (95% CI, 0.57–0.91; *p* = 0.006) [62].

#### 5.1.2. Relapsed or Refractory cHL

##### Second Line Treatment

**Figure 3:**
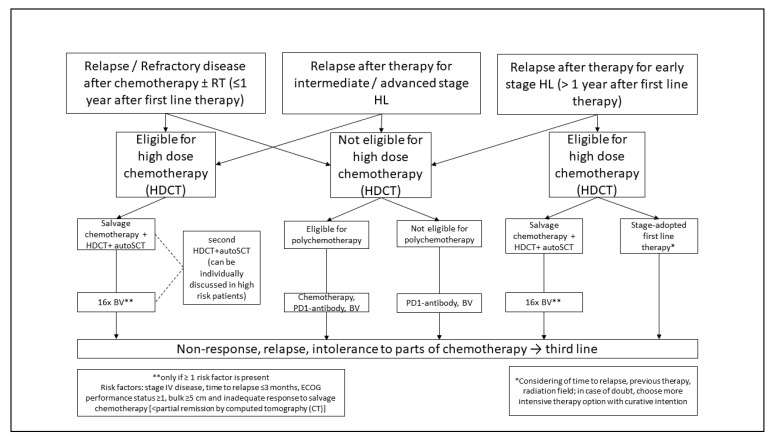
Therapy algorithm for relapsed/refractory HL.

Following front-line treatment failure—i.e., histopathological proven refractory or relapsed disease—the application of an intensive salvage chemotherapy followed by high-dose chemotherapy (HDCT) and autologous stem cell transplant (ASCT) is regarded as the standard of care [Figure 3]. This approach results in a cure rate of about 50% [63,64,65]. Salvage treatment applied before HDCT and ASCT usually includes two cycles of platinum- or gemcitabine-based salvage regimen and is administered to achieve a good response before ASCT as well as to mobilize sufficient bone marrow stem cells. With the most commonly used second line regimens such as DHAP (dexamethasone, high-dose cytarabine, cisplatin), ICE (ifosfamide, carboplatin, etoposide), IGEV (ifosfamide, gemcitabine, vinorelbine, prednisone), GDP (gemcitabine, dexamethasone, cisplatin), GVD (gemcitabine, vinorelbine, liposomal doxorubicin) or ESHAP (etoposide, methylprednisolone, high-dose cytarabine, cisplatin) response rates of 70–80% and a CR (complete remission) rate of 20–50% has been achieved in single-arm phase II trials. No superiority of one over the other regimens has been demonstrated. For patients achieving PET-negativity after salvage chemotherapy, a good outcome with relapse rates of 15–30% after ASCT has been reported, whereas the chance of cure is significantly lower in patients with a positive PET/CT before high-dose chemotherapy and ASCT [66]. In addition to the response in second line chemotherapy primary refractory disease, stage IV disease at relapse, ECOG-status 1 and a nodal lesion > 5 cm at relapse were identified as independent relevant risk factors for the outcome after ASCT in a large multivariate analysis [67].

The prognostic relevance of PET-negativity before ASCT indicates that more effective salvage regimen might improve the outcome after HDCT and ASCT. Promising new salvage approaches include BV either in combination with conventional platinum- or gemcitabine-based chemotherapy or with bendamustine or within a sequential, response-adapted strategy [68,69,70]. The combination of BV and the anti-PD1 checkpoint inhibitors Nivolumab or Pembrolizumab result in a significantly increased rate of complete metabolic response and an improved outcome after ASCT providing also a chemotherapy-free and thus less toxic salvage regimen [71].

A different established approach to improve the results of HDCT and ASCT is the application of consolidating treatment with BV in high-risk patients. In the AETHERA trial, consolidating the application of 16 infusions of BV after ASCT resulted in a significant improvement of disease control in patients with either primary refractory disease, early relapse (<12 months) or extranodal relapse [72]. Based on these results, BV was approved as consolidation therapy for relapsed/refractory (r/r) cHL patients with increased risk of relapse after ASCT. High risk patients may benefit from tandem ASCT.

In most of r/r cHL patients who are not eligible for ASCT due to age, comorbidity, poor performance status or progressive disease on second-line treatment, the outcome is even poorer since the response rates and duration of response achieved with conventional second line chemotherapy are disappointing [73]. Thus, there is a need to implement targeted or immunomodulatory treatment approaches in the second line treatment of this patient cohort (please refer to Section 5.3).

##### Relapse After Second Line Treatment

In the majority of patients with disease recurrence after HDCT and ASCT, a chemotherapy-refractory disease has to be assumed; treatment options are therefore limited and the reported estimated median overall survival achieved with a conventional treatment is only 2–3 years [74]. With the approval of the anti-CD30 immunoconjugate BV and the anti-PD1 antibodies Nivolumab and Pembrolizumab, new treatment options have become available and resulted in high response rates even in chemotherapy-refractory and heavily pretreated cHL patients.

In the pivotal phase II trial, BV resulted in a response rate of 75% and a median PFS of 9.3 months in cHL patients relapsing after ASCT [75]. In patients achieving a complete response with BV, 34% of these patients treated in a phase II trial, a 5-year PFS of 52% was documented indicating a potentially curative role of BV in a minority of patients with r/r cHL after ASCT [76]. The real life data are consistent with the reported pivotal phase II trial, including comparable response rates in those patients who are ineligible for ASCT due to advanced age or comorbidity [77].

Whether BV or an anti-PD-1 antibody should be used in case of a relapse after ASCT was addressed in the Keynote-204 trial (NCT02684292). It included patients with relapse after ASCT and patients who were not suitable for ASCT. For the overall group, there was a statistically significant better PFS for pembrolizumab of 13.2 vs. 8.3 months. Although the results of the study have not yet been available as a full text publication and Pembrolizumab is not yet approved by the EMA for this indication, the results seem so convincing that Pembrolizumab should be discussed as a new standard for patients with relapse after autologous SCT [78].

Due to a limited duration of response in the majority of patients treated with BV, there was still an urgent need to develop additional new treatment approaches. The idea to reactivate exhausted T-lymphocytes in the microenvironment of cHL by blockade of the checkpoint molecule PD1 revolutionized the treatment of r/r cHL: the application of Nivolumab and Pembrolizumab in the pivotal phase I/II trials resulted in response rates of about 70% and a median PFS of 13–15 months in patients with prior ASCT and Brentuximab [79,80]. Although the rate of complete responses achieved with Nivolumab and Pembrolizumab in these trials was rather low and most patients relapsed within the follow-up period, excellent overall survival rates of over 80% after 2 years could be documented. These data as well as subgroup analyses in patients receiving treatment beyond progression and retrospective analyses of the effect of subsequent lines of therapy after anti-PD1 blockade indicate that the immunomodulatory approach of PD1-blockade might have a significant impact on the biology of r/r cHL [81,82].

With the introduction of anti-PD1 antibodies in the treatment of r/r cHL the role and the optimal timing of allogeneic stem cell transplant have been questioned. The best results so far with 4-year PFS- and OS rates of about 50% in r/r cHL have been achieved with haploidentical transplantation and GvHD prophylaxis with post-transplant cyclophosphamide [83]. With regard to the excellent OS rates achieved with anti-PD1 blockade more clinical data need to be generated to identify those patients with the best benefit of allogeneic SCT.

### 5.2. Nodular Lymphocyte-Predominant HL (NLPHL)

#### 5.2.1. First Line Treatment

Nodular lymphocyte-predominant HL (NLPHL) accounts for approximately 5% of all HL cases. Pathological and clinical characteristics differ from cHL. Histopathologically, the malignant lymphocyte predominant cells in NLPHL are consistently positive for CD20, but lack CD30. Clinically, most cases are diagnosed in early stages and the course is usually indolent. However, a tendency towards late relapses and histological transformation into aggressive B-cell non-Hodgkin lymphoma (B-NHL) has been described [84].

At most institutions, the standard treatment for stage IA NLPHL without clinical risk factors consists of limited-field RT alone. Different retrospective studies have demonstrated that the addition of chemotherapy does not further improve the results obtained with RT alone [85,86]. Early favorable-stage disease other than stage IA without clinical risk factors is usually treated with two cycles of chemotherapy followed by limited-field RT. Disease control with this approach appears to be better than with RT alone [87]. The chemotherapy protocol most frequently used is ABVD. Patients with early unfavorable-stage NLPHL also receive combined-modality treatment (CMT) in the majority of cases. In this situation, a total of four cycles of ABVD followed by limited-field RT are mostly applied [88]. The question of whether anti-CD20 antibodies should be included in the first-line treatment of early favorable and early unfavorable-stage NLPHL has been unanswered so far. The treatment options for advanced NLPHL include interim-PET-guided eBEACOPP, R-CHOP (rituximab, cyclophosphamide, doxorubicin, vincristine, prednisone) and BR (bendamustine, rituximab) [89,90,91]. In contrast to these regimens, ABVD appears to be associated with poorer disease control [92]. The optimal approach for the individual patient suffering from advanced NLPHL should be chosen on the basis of different factors including the patient’s age, the extent of the disease at diagnosis and the presence or absence of systemic symptoms. Overall, patients with NLPHL have an excellent outcome after stage-adapted first-line treatment. Individuals with stage IA disease without clinical risk factors have a 10-year PFS rate of roughly 90% and a 10-year OS rate close to 100% [86]. The 10-year PFS and OS rates for patients with more advanced disease range from 70% to 80% and 88% to 96%, respectively [88].

#### 5.2.2. Relapsed NLPHL

There is no standard approach for the treatment of relapsed NLPHL. Once histological transformation into aggressive B-NHL has been ruled out and the diagnosis of NLPHL has been confirmed, the treatment is chosen individually based on factors such as patient’s age, previous treatment, stage at relapse and lymphoma-related symptoms. Given the mostly indolent course of the disease even in case of recurrence, most patients do not require aggressive salvage therapy with high-dose chemotherapy and autologous stem cell transplantation, but are treated sufficiently with conventional chemotherapy optionally combined with an anti-CD20 antibody and/or RT, RT alone or single-agent anti-CD20 antibody treatment. According to the results of a retrospective study including 99 patients with relapsed NLPHL who had received different salvage therapies, the 5-year PFS and OS rates after lymphoma recurrence were 75.6% and 89.5%, respectively [93]. These results are consistent with additional analyses also investigating the outcome of patients with relapsed NLPHL [94,95].

Future studies and analyses including patients with NLPHL should aim at identifying individuals with a high risk for an aggressive course and thus requiring intensive treatment. Low-risk patients should receive nontoxic approaches to avoid treatment-related late effects whenever possible. Such approaches may include low-dose conventional chemotherapy in combination with an anti-CD20 antibody.

### 5.3. Treatment of Elderly Patients

In cancer registry studies, the rate of patients over 60 years is considered 25% of all patients with Hodgkin lymphoma [7,96]. The successful progress in treatment results in young patients (<60 years) could not be translated to the elderly patient cohort [97]. The reasons for the poorer treatment results in the elderly cohort are diverse: for one thing, elderly patients usually do not tolerate intensive chemotherapy regimens, for example due to concomitant diseases and impaired organ function. Additionally, the course of the disease in older patients often appears more aggressive than in the younger patient cohort [98,99].

Thus, the status of these patients should be carefully assessed beforehand. Within the SHIELD study, a modified ACE-27(“adult comorbidity evaluation-27”) score was used, to detect frail patients with a higher risk for complications during therapy. Those who are classified as frail did not receive multiagent chemotherapy. Despite only including a small number of cases, the trial showed that an objective description of the state of health helps to assess the ability to treat [92]. Andrew M. Evens and colleagues assessed a connection between the success of the therapy and age, as well as the ability to do everyday activities: the geriatric evaluation of the patients who were treated for Hodgkin lymphoma was performed using CIRS-G (“cumulative illness rating scale-geriatric”) score. Two factors seem to impair outcome for elderly patients who had a negative impact on PFS and OS: age (>70 years) and a loss of activities of daily life (ADL) [100].

Based on the available trial data elderly patients with early-stage favorable cHL and eligible for chemotherapy should be treated with two cycles of ABVD and additional 20 Gy radiotherapy similar to the younger patient cohort [101]. Early-unfavorable cHL in patients older than 60 years should be treated with two cycles of ABVD and two cycles of AVD with consecutive radiotherapy ad 30 Gy ISRT [73].

Due to toxicity, elderly patients should not be treated with more than two cycles of ABVD including Bleomycin due to the higher risk for long term lung toxicity or eBEACOPP [99]. Instead, treatment with two cycles ABVD and a further four cycles of AVD and subsequent irradiation of PET-positive residues > 1.5 cm can be regarded as the treatment of choice. A more recent phase II study using PVAG (prednisone, vinblastine, doxorubicin, gemcitabine) for 59 patients in the intermediate and advanced stages showed 3-year overall survival rates (OS) of 66% (95% CI, 50–78) and 3-year PFS rates of 58% (95% CI, 43–71). Thus, PVAG could be considered a Bleomycin-free alternative with similar efficacy and tolerability as ABVD [102].

## 6. Future Treatment Approaches

The introduction of Brentuximab Vedotin and PD-1 inhibitors such as Pembrolizumab and Nivolumab has opened up new treatment options for Hodgkin’s lymphoma [75,79,80]. Studies in relapsed or refractory Hodgkin’s lymphoma resulted in excellent response rates and long-lasting responses with an acceptable side effect profile could be observed in a small percentage of patients. Consequently, the introduction of these drugs in first-line therapy will become a feasible option in future trials.

In the randomized Phase II NIVAHL trial conducted by the GHSG, 109 patients aged 18–60 years and diagnosed with intermediate stage cHL were included and treated with a total of eight doses of Nivolumab and four cycles of AVD and subsequent consolidating 30Gy ISRT, respectively. In arm A, Nivolumab and AVD were administered simultaneously; in arm B, patients received four doses of Nivolumab upfront, followed by two cycles of Nivolumab-AVD and two cycles of AVD. The results were rather promising: after two cycles of Nivolumab + AVD or after four Nivolumab doses, a CR could be documented in 47/54 (87%) and 26/51 (51%) of all cases. At the end of the systemic therapy, the CR rate was 90% (46/51) and 94% (47/50) in all evaluable patients, respectively. With a median follow-up time of 14 (6–27) months for arm A and 13 (8–28) months for Arm B, a PFS rate of 100% and 98% was documented [103].

Similar to the NIVAHL study, the cohort D of the CheckMate 205-trial enrolled newly diagnosed patients with an advanced stage of cHL. These patients were treated with a combination of Nivolumab and AVD: patients received four doses of Nivolumab initially followed by 12 additional doses of Nivolumab in combination with six cycles of AVD. Of note: six patients (range 61–87) over the age of 60 were also enrolled. After four doses of Nivolumab, a response rate of 69% (35/51) was documented, and 18% (9/51) of the patients achieved a CR. After completion of the systemic therapy, 46/51 (90%) patients showed a response with 34/51 (67%) achieving a CR. Progressive disease was only documented in two cases. Interestingly five of the six elderly patients included in this trial achieved a CR after the completion of therapy [104]. However, data from a randomized phase III trial is lacking for both concepts.

The introduction of checkpoint inhibitors (Cis) and their success in the treatment of relapsed/refractory cHL suggest that an implementation of these drugs in first line therapy approaches enables new, less toxic and effective treatment strategies in the near future.

## Figures and Tables

**Table 1:** Ann Arbor classification for Hodgkin’s lymphoma.

Stage	Explanation
I	Involvement of one lymph node region or a single localized involvement outside the lymphatic system
II	Involvement of two or more lymph node regions on the same side of the diaphragm or localized involvement outside the lymphatic system and lymph node regions on the same side of the diaphragm
III	Involvement of two or more lymph node regions or organs outside the lymphatic system on both sides of the diaphragm
IV	Diffuse or disseminated infestation of one or more extralymphatic organs with or without infestation of lymphoid tissue
A	No B-symptoms
B	B symptoms: fever, drenching night sweats, and/or unexplained loss of body weight >10% within the preceding 6 months.

**Table 2:** Risk factor definitions in Hodgkin’s lymphoma. Patients in CS I–II are staged unfavorable if at least one of the listed risk factors is present [34].

Risk Factors	GHSG	EORTC	NCIC/ECOG	NCCN
Large mediastinal mass	Yes ^1^, ratio ≥ 1/3	Yes, ratio ≥ 0.35	No	Yes, ratio > 1/3
Extranodal disease	Yes ^1^	No	No	Yes
Nodal areas	Yes, ≥3 areas	Yes, ≥4 areas	Yes, ≥4 areas	Yes, ≥3 regions
ESR	Yes, ≥50 (A) or ≥30 (B)	Yes, ≥50 (A) or ≥30 (B)	Yes, ≥50	Yes, ≥50 (A)
B-symptoms	No	No	No	Yes
Bulk	No	No	No	Yes, >10 cm
Age	No	Yes, ≥50 years	Yes, ≥40 years	No
Histology other than LP/NS	No	No	Yes	No

Abbreviations: CS clinical stage, ESR erythrocyte sedimentation rate, A without B-symptoms, B with B-symptoms, LP lymphocyte predominant, NS nodular sclerosis, GHSG German Hodgkin Study Group, EORTC European Organization for Research and Treatment of Cancer, NCIC National Cancer. Institute of Canada, ECOG Eastern Cooperative Oncology Group, NCCN National Comprehensive Cancer Network. ^1^ According to GHSG, patients with CSIIB and a large mediastinal mass or extranodal disease are considered as advanced stage.

## Data Availability

Not applicable.
